# Assessment of Fatty Acid Profile, Mineral Composition, and Antioxidant Activity of Fermented Whey Beverages from Organic Cow and Goat Whey with the Organic Sea Buckthorn or Rosehip Juices

**DOI:** 10.3390/molecules31111905

**Published:** 2026-06-01

**Authors:** Maciej Bartoń, Anna Stępniowska, Katarzyna Ognik, Bartosz G. Sołowiej

**Affiliations:** 1 Department of Dairy Technology and Functional Foods, Faculty of Food Sciences and Biotechnology, University of Life Sciences in Lublin, Skromna 8, 20-704 Lublin, Poland; maciej.barton@up.edu.pl; 2 Department of Biochemistry and Toxicology, Faculty of Animal Sciences and Bioeconomy, University of Life Sciences in Lublin, Akademicka 13, 20-950 Lublin, Poland; anna.stepniowska@up.edu.pl (A.S.); katarzyna.ognik@up.edu.pl (K.O.)

**Keywords:** fermented whey beverages, polyphenols, minerals and trace elements, fatty acid methyl esters, functional foods

## Abstract

This study examined the chemical composition and functional properties of fermented whey beverages produced from organic cow and goat whey, including both acid and sweet variants, enriched with organic sea buckthorn (*Hippophae rhamnoides*) or rosehip (*Rosa canina*) juices. In contrast to earlier research primarily addressing physicochemical and technological aspects, the present work offers a comprehensive evaluation of fatty acid composition, mineral and trace element content, antioxidant activity, and total polyphenol levels in these beverage formulations. Both the type of whey and the fruit additive significantly influenced the compositional profile and antioxidant capacity of the beverages. Samples fortified with rosehip demonstrated the highest antioxidant potential, as evidenced by enhanced DPPH radical scavenging activity, elevated FRAP values, and increased total polyphenol content. In comparison, beverages enriched with sea buckthorn juice exhibited higher concentrations of selected minerals, particularly Fe and Ni, while maintaining toxic element levels within permissible limits. The fatty acid profile was predominantly composed of saturated fatty acids, notably C16:0, along with short-chain fatty acids typical of whey. Beverages derived from goat whey contained higher levels of SFA and MUFA than those produced from cow whey, whereas the addition of rosehip contributed to increased proportions of PUFA and omega-3 fatty acids. Collectively, these findings indicate that organic fermented whey beverages enriched with rosehip or sea buckthorn juice may serve as promising functional products with enhanced antioxidant properties and favorable mineral and fatty acid profiles.

## 1. Introduction

Fermented dairy beverages have attracted growing scientific and industrial interest in recent years because of their functional potential, bioactive properties, and relevance to sustainable food production and circular economy principles [1,2,3,4,5]. Among various substrates used for their manufacture, whey—a by-product of cheese production—remains an underutilized yet valuable material rich in high-quality proteins, bioactive peptides, essential amino acids, lactose, and minerals [1,2,3]. Utilizing whey in the form of fermented beverages may help reduce environmental burdens associated with whey disposal while expanding the portfolio of functional products within the dairy industry [1,5,6,7]. Organic cow and goat whey are particularly attractive raw materials for minimally processed beverage formulations produced without synthetic additives. Goat whey, characterized by higher concentrations of short- and medium-chain fatty acids, exhibits superior digestibility, faster energy release, and distinct sensory and nutritional properties compared with cow whey [2,3]. Recent advances in functional beverage development have focused on enriching dairy matrices with plant-derived bioactive compounds, particularly fruit juices abundant in polyphenols, carotenoids, and vitamin C. Among these, sea buckthorn (*Hippophae rhamnoides*) and rosehip (*Rosa canina*) are promising fruit ingredients due to their high content of polyphenols, carotenoids, vitamin C, and lipid-soluble bioactive compounds [7,8]. Incorporating such fruit components into fermented whey beverages not only enhances antioxidant activity, sensory attributes, and consumer appeal but also improves the nutritional and functional profile of the final product [9,10]. Furthermore, minerals and trace elements as calcium, iron, zinc, and selenium contribute to key metabolic and immune processes, while low levels of toxic elements ensure product safety in accordance with European Union food safety regulations concerning contaminants in food [11,12]. The antioxidant capacity of fermented whey beverages—typically evaluated using DPPH, ABTS, and FRAP assays—serves as a reliable indicator of their bioactivity and oxidative stability [1,5,6]. A similarly comprehensive analytical approach, combining physicochemical, rheological, microbiological and antioxidant assessments of fermented functional beverages, was reported by Salek et al. [13], who demonstrated that fermentation with symbiotic starter cultures significantly enhances bioactive potential and oxidative stability. The integration of organic whey with functional fruit ingredients such as sea buckthorn and rosehip (*R. canina*) thus presents a promising strategy for developing sustainable, health-promoting beverages with improved physicochemical and bioactive characteristics.

Among the plant-based additives considered for the fortification of fermented whey beverages, *R. canina* and *H. rhamnoides* stand out as particularly rich natural sources of bioactive compounds. *R. canina* fruits contain abundant phenolic acids, flavonoids, carotenoids, tocopherols, and vitamin C, which collectively contribute to their strong antioxidant, anti-inflammatory, and hepatoprotective activities [14]. Concentrations of ascorbic acid as high as 419.7 mg/100 g DW, along with substantial levels of carotenoids and polyphenols identified across different ecotypes, highlight the exceptional antioxidant capacity of this species [15]. Furthermore, encapsulated or powdered rosehip preparations effectively retain vitamins and minerals, making them valuable functional ingredients in fruit-enriched or dairy-based beverages [16]. In a whey-based matrix, synergistic interactions among flavonoids, carotenoids, vitamin C, and whey-derived proteins or peptides may be relevant because they can influence antioxidant activity, oxidative stability, and the functional quality of fermented beverages [17].

Similarly, *H. rhamnoides* berries are recognized as a potent reservoir of bioactive constituents, including carotenoids, tocopherols, phenolic acids, and unsaturated fatty acids, alongside exceptionally high levels of vitamin C—reported to reach up to 2740 mg/100 g [18]. Incorporation of sea buckthorn into food systems enhances nutritional value, sensory quality, and antioxidant activity, aligning with principles of sustainable and circular food production [19]. Recent developments in eco-efficient extraction technologies, such as ultrasound-assisted methods, have further improved the recovery of phenolic compounds, carotenoids, and essential fatty acids, reinforcing the multifunctional potential of this plant in nutraceutical and food applications [20]. Additionally, dairy products or beverages enriched with sea buckthorn pulp or oil exhibit improved oxidative stability and nutrient retention, thereby enhancing both the functional and technological attributes of fermented milk matrices [21]. In this context, sea buckthorn emerges as a promising raw material for innovative fermented food products, enabling the simultaneous improvement of nutritional quality, oxidative stability, and technological performance [22].

This study builds upon and functionally extends our previous research on organic fermented whey beverages fortified with sea buckthorn or rosehip juice. In the earlier investigation, we assessed key technological and sensory-related properties of comparable beverages, including apparent and dynamic viscosity, rheological and viscoelastic characteristics, pH, titratable acidity, and microbiological safety. These parameters are intrinsically linked to texture, mouthfeel, product stability, and overall technological suitability. The current study advances this framework by examining the fatty acid profile, mineral and trace element composition, total polyphenol content, and antioxidant activity.

Despite the growing interest in fermented whey beverages enriched with fruit-derived bioactive compounds, most existing studies have predominantly focused on physicochemical, rheological, microbiological, or general antioxidant characteristics. In contrast, there is a lack of comprehensive data addressing the combined effects of whey origin, whey type, and fruit juice supplementation on fatty acid composition, mineral and trace element content, total polyphenol levels, and antioxidant activity in organic fermented whey beverages. Accordingly, the present study builds upon and functionally extends our earlier work on organic fermented whey beverages enriched with sea buckthorn or rosehip juice, in which key technological and sensory-related parameters—such as viscosity, rheological and viscoelastic properties, pH, titratable acidity, and microbiological safety—were evaluated.

Therefore, the objective of this study was to assess the fatty acid composition, mineral and trace element profile, total polyphenol content, and antioxidant activity of fermented beverages produced from organic cow and goat whey, including both acid and sweet whey, fortified with sea buckthorn or rosehip juice. This work contributes to the ongoing research on dairy-based functional foods by providing new insights into how whey type and plant-based fortification influence the bioactive composition and nutritional quality of these products.

## 2. Results and Discussion

### 2.1. Antioxidant Activity and Polyphenolic Profile of Fermented Whey Beverages Enriched with R. canina and H. rhamnoides

Among the plant-derived ingredients incorporated into fermented whey beverages, *R. canina* and *H. rhamnoides* are recognized as outstanding sources of natural antioxidants. In the present study, samples fortified with rosehip juice—specifically SKK/DR, SKKr/DR, SSK/DR, and SSKr/DR—show the strongest antioxidant performance, with DPPH inhibition values ranging from 81 to 95%, ABTS inhibition ranging from 33 to 42%, FRAP levels between 0.98 and 1.16 mmol TE/L, and total polyphenol contents reaching 327–422 mg/100 g. In contrast, NP and samples fortified with sea buckthorn juice samples, namely SKK/NP, SKKr/NP, SSK/NP, SSKr/NP, SKK/R, SKKr/R, and SSK/R, exhibit the lowest antioxidant potential, with DPPH inhibition values ranging from 3.01 to 61.57%, ABTS inhibition between 3.01 and 29.55%, FRAP values not exceeding 0.77 mmol TE/L, and total polyphenol contents of 21.2–41.9 mg/100 g. These findings are consistent with earlier research indicating that *R. canina* fruits are rich in phenolic acids, flavonoids, carotenoids, tocopherols, and vitamin C, all of which contribute to substantial antioxidant activity [23]. The antioxidant properties and polyphenol content of the analyzed fermented whey beverages are presented in Table 1.

Numerous studies have emphasized strong correlations between total polyphenol levels and antioxidant capacity in rosehip-based formulations, confirming that phenolic compounds largely determine the reducing and radical-scavenging power of the product [24]. Synergistic effects among flavonoids, carotenoids, and ascorbic acid have also been shown to enhance oxidative stability, thereby strengthening the antioxidant potential of rosehip extracts. Moreover, fermentation processes may further intensify polyphenol content and antioxidant response, as demonstrated in microbial transformations of *Rosa rugosa*, where both DPPH and FRAP increased following fermentation [25].

Sea buckthorn whey beverages analyzed in this study also exhibit notable antioxidant properties. Across all *H. rhamnoides* samples (R variants), DPPH inhibition ranges from 41.52 to 94.62%, ABTS inhibition ranges from 3.01 to 19.05%, and FRAP values range from 0.519 to 0.815 mmol TE/L. The highest FRAP value among beverages enriched with sea buckthorn juice is observed in samples of SKKr/R (0.815 mmol TE/L). These observations agree with reports describing the robust antioxidant potential of *H. rhamnoides,* attributed to its abundant polyphenols, flavonoids, and carotenoids acting through hydrogen-donating and electron-transfer mechanisms [26]. The results obtained from DPPH, ABTS, and FRAP analyses correspond with literature showing strong positive correlations between total phenolic content and antioxidant capacity in sea buckthorn juices and their derivatives [27]. Similarly, probiotic or co-fermented beverages containing *H. rhamnoides* have demonstrated DPPH inhibition exceeding 90% and elevated polyphenol levels, indicating that controlled fermentation can further enhance the functional potential of fruit-based beverages [28].

The differences between beverages enriched with rosehip juice and those enriched with sea buckthorn juice likely stem from the distinct bioactive compositions of these fruits. Rosehip supplementation results in higher total polyphenol concentrations and stronger reducing power, whereas sea buckthorn addition provides balanced radical-scavenging activity and moderate FRAP values, reflecting its complex carotenoid–polyphenol matrix [20].

### 2.2. Analysis of Antioxidant Indices Ratios and Their Interpretation in Relation to the Polyphenolic Profile of Fermented Beverages Enriched with R. canina and H. rhamnoides

Ratio analysis of antioxidant indices reveals distinct antioxidant behaviors between the beverages enriched with *R. canina* and *H. rhamnoides*. The highest DPPH/FRAP values (108.3 ± 43.4) are observed in sea buckthorn beverages, indicating that the radical-scavenging activity measured by DPPH is relatively high compared with the reducing power measured by FRAP. This pattern suggests that *H. rhamnoides* beverages contain antioxidant compounds with strong hydrogen-donating capacity but a more moderate electron-transfer potential. Such characteristics are consistent with the known activity of sea buckthorn flavonoids and carotenoids, which participate in radical quenching through hydrogen atom transfer mechanisms [29]. The ratios between antioxidant indices for fermented whey beverages enriched with *R. canina* and *H*. *rhamnoides* are presented in Table 2.

In contrast, the rosehip beverages display much lower DPPH/FRAP ratios (74.3 ± 21.9) and exceptionally low FRAP/ABTS values (0.029 ± 0.003), indicating that *R. canina* polyphenols exert stronger reducing capacity relative to their ABTS and DPPH radical scavenging responses. This finding aligns with previous evidence demonstrating that gallic acid, catechin, quercetin, and other phenolics abundant in rosehip exhibit high electron-donating potential and strong reducing power [30].

The TPC/DPPH ratio further differentiates the two fruit matrices. Rosehip beverages exhibit a markedly higher TPC/DPPH value (4.9 ± 1.1) compared with sea buckthorn beverages (0.7 ± 0.3), highlighting the substantially greater contribution of total polyphenols to antioxidant activity in *R. canina* formulations. This observation aligns with earlier studies confirming strong correlations between phenolic abundance and antioxidant activity in rosehip-derived systems [31]. In contrast, the lower TPC/DPPH ratio in sea buckthorn beverages suggests that non-phenolic antioxidants, such as carotenoids and vitamin C, may contribute more significantly to their radical-scavenging efficiency.

Strong correlations between total polyphenol content and antioxidant capacity in the present study reinforce the view that phenolic compounds remain the principal determinants of antioxidant performance in fermented matrices enriched with *R. canina* and *H. rhamnoides*. Similar associations have been reported in whey and yogurt systems fortified with plant-derived bioactive compounds [4].

The distinct antioxidant behaviors of rosehip and sea buckthorn beverages reflect the differences in their bioactive profiles. Rosehip supplementation leads to higher polyphenol concentrations and greater reducing power, whereas sea buckthorn addition results in stronger DPPH/FRAP responses and more balanced radical-scavenging mechanisms, consistent with its complex carotenoid–flavonoid composition [20]. These findings agree with previous research demonstrating complementary antioxidant mechanisms arising from the interaction of polyphenols, carotenoids, and vitamin C in *H. rhamnoides* [29].

Further supporting evidence indicates that ultrasonic and enzymatic extraction techniques enhance the recovery of bioactive compounds from *R. canina* and *H. rhamnoides*, resulting in improved antioxidant yields in fermented systems [32]. The synergistic interactions among polyphenols, carotenoids, and ascorbic acid contribute to the oxidative stability of these beverages, reinforcing their functional potential [33].

### 2.3. Fatty Acid Composition of Fermented Whey Beverages Enriched with R. canina and H. rhamnoides

The fatty acid profiles of fermented whey beverages enriched with *R. canina* and *H. rhamnoides* display distinct modifications determined by both the type of fruit juice and the origin of the whey matrix. In all samples, saturated fatty acids (SFAs) represent the dominant fraction, mainly palmitic acid (C16:0) together with short-chain fatty acids (C4:0–C10:0) typical of dairy-derived lipids. Nevertheless, supplementation with rosehip or sea buckthorn juice significantly modifies the lipid composition by increasing the proportions of unsaturated and polyunsaturated fatty acids (PUFA), thereby improving both the nutritional quality and functional value of the beverages.

To enhance the nutritional interpretation of the fatty acid profile, the n−6/n−3 ratio was calculated and is reported in Table 3. This ratio exhibits substantial variability among the samples, reflecting the combined influence of whey origin, whey type, and fruit juice addition. The lowest values are recorded for SKKr/DR and SKKr/NP, at 0.67 and 0.82, respectively. In several beverages based on goat whey and those enriched with rosehip juice, the ratio ranges from 1.36 to 1.58, suggesting a relatively balanced proportion of omega-6 to omega-3 fatty acids. In contrast, beverages enriched with sea buckthorn juice generally display higher n−6/n−3 ratios, ranging from 4.43 to 5.73, while pure sea buckthorn juice reaches a value of 12.40. For the SKK/DR sample, the ratio is not determined, due to the absence of detectable omega-3 fatty acids.

Beverages fortified with *R. canina* juice—particularly SKK/DR, SKKr/DR, and SSKr/DR—show elevated PUFA content, with linoleic (C18:2 n−6) and α-linolenic (C18:3 n−3) acids as the predominant components. A comparison between SKK/DR and its cow whey equivalent SKKr/DR indicates that the acid goat whey matrix may also contribute to the increased PUFA content. SKK/DR contains 0.089 g/100 g PUFA, whereas SKKr/DR contains 0.015 g/100 g PUFA, despite both samples being enriched with rosehip juice. This suggests that the final PUFA level is determined not only by the fruit additive but also by the whey origin and its interaction with the fruit-derived lipid fraction. This composition mirrors the fatty acid profile of rosehip oil, which typically contains 45–55% linoleic acid and 20–30% α-linolenic acid, contributing to a favorable omega-6/omega-3 balance and supporting cardiovascular and anti-inflammatory functions. The incorporation of rosehip juice thus contributes to the enrichment of the lipid fraction of fermented whey with essential fatty acids, supporting the bioactive potential of the resulting beverages [21].

In samples containing *H. rhamnoides* juice, the lipid profiles are characterized by increased levels of monounsaturated fatty acids (MUFAs), notably palmitoleic (C16:1 n7) and oleic (C18:1 n9) acids. These compounds are well-established markers of sea buckthorn lipids derived from seed and pulp fractions, known for their roles in metabolic regulation and tissue regeneration. The presence of palmitoleic acid (omega-7) is particularly important, as it exhibits anti-inflammatory and regenerative activities that complement the antioxidant mechanisms of sea buckthorn polyphenols. Comparable fatty acid distributions, distinguished by high MUFA and moderate PUFA levels, have been reported for diverse sea buckthorn genotypes, confirming its value as a plant source of bioactive lipids [11]. Moreover, compositional studies indicate that sea buckthorn berries may contain up to 76% C18 fatty acids, dominated by linoleic acid (29–50%) within the polyunsaturated fraction [20].

The origin of the whey matrix further influences the final lipid composition. Beverages prepared from goat whey contain greater quantities of short- and medium-chain fatty acids, including butyric (C4:0), caproic (C6:0), and capric (C10:0) acids, which are associated with antimicrobial and gut-health benefits. In contrast, cow whey-based beverages display higher proportions of long-chain unsaturated fatty acids. These differences reflect the metabolic distinctions between milk sources and the specific interactions among whey proteins, microbial cultures, and fruit lipids during fermentation [1].

From a functional standpoint, the incorporation of *R. canina* and *H. rhamnoides* juices into the fermented whey system contributes to a more diverse lipid profile that combines dairy-derived fatty acids with bioactive plant lipid fractions. The presence of omega-3, omega-6, and omega-7 fatty acids supports the nutritional relevance of these beverages; however, their interpretation should consider the calculated n−6/n−3 ratio presented in Table 3 (Part b). These outcomes align with recent advances in functional dairy research, where the inclusion of sea buckthorn and rosehip lipids has been shown to enhance both the nutraceutical value and oxidative stability of fermented milk products [21].

### 2.4. Heavy Metal Content in Fermented Whey Beverages Enriched with R. canina and H. rhamnoides

The determination of heavy metal content in fermented whey beverages enriched with *R. canina* and *H. rhamnoides* juices demonstrates that all toxic elements—arsenic (As), cadmium (Cd), lead (Pb), and mercury (Hg)—remain well below the permissible limits established by European food safety regulations, confirming the toxicological safety and ecological integrity of the products. Among the analyzed elements, iron (Fe), zinc (Zn), and manganese (Mn) predominated in the mineral fraction, whereas toxic metals are detected only in trace concentrations. Measured As levels range from 0.004 to 0.016 mg/kg, Cd from <0.001 to 0.007 mg/kg, and Pb from 0.032 to 0.11 µg/kg, while Hg concentrations remain below the quantification limit. These values are significantly lower than the maximum levels allowed under Commission Regulation (EC) No. 1881/2006, as amended by Commission Regulation (EU) 2023/915, which stipulate threshold concentrations of Cd (≤0.01 mg/kg) and Pb (≤0.02 mg/kg) for milk and dairy products [12,34].

Sea buckthorn beverages show generally higher Fe and Ni contents, whereas Mn levels are highly elevated in pure rosehip juice, reflecting natural variability in fruit mineral profiles. This trend reflects the natural mineral composition of sea buckthorn fruit, known for its abundance of essential transition metals serving as cofactors in antioxidant enzymes such as superoxide dismutase and catalase. Previous research has shown that despite relatively high Fe and Mn concentrations in sea buckthorn biomass, Cd, Hg, and Pb levels remain well below EU safety thresholds, highlighting the species’ low capacity for heavy metal bioaccumulation [35]. Furthermore, the incorporation of sea buckthorn into fermented dairy matrices has been linked to enhanced antioxidant stability and nutritional quality without elevating heavy metal content [4,7].

Conversely, beverages fortified with *R. canina* juice (SKK/DR, SKKr/DR, Rosehip juice) exhibit slightly lower Fe and Mn levels but comparable or marginally higher As and Cd concentrations. This distribution aligns with the intrinsic mineral composition of rosehip fruits, characterized by balanced levels of Fe, Cu, and Zn, and minimal amounts of toxic trace elements. The high polyphenol and carotenoid content typical of *R. canina* contributes to pronounced metal-chelating activity, facilitating the binding or immobilization of trace metals during fermentation and storage [36].

In general, essential elements such as Fe, Zn, and Mn predominate over toxic elements, although their exact distribution varies depending on whey origin, whey type, and fruit juice addition. This sample-specific variability is particularly evident in rosehip-based samples and pure rosehip juice, where Mn levels are elevated compared with other variants. Overall, the predominance of essential elements and the trace-level presence of toxic metals are consistent with patterns reported for other fermented dairy and plant-based beverages [37]. These findings confirm that the fermentation process and supplementation with *R. canina* or *H. rhamnoides* juices did not promote heavy metal accumulation. Moreover, the trace levels of toxic elements are consistent with those reported for functional dairy products containing sea buckthorn and rosehip, all of which comply with current EU food safety and environmental sustainability requirements [33].

From a nutritional and functional standpoint, the presence of Fe, Mn, and Zn augments the biological value of fermented whey beverages by supporting antioxidant enzymatic systems and maintaining redox balance. These beneficial microelements coexist with negligible amounts of toxic metals, confirming that plant juice supplementation and fermentation improved the nutritional quality of the beverages without compromising safety. The antioxidant and metal-chelating activities of *R. canina* and *H. rhamnoides* further stabilize trace element composition, ensuring both functional efficacy and toxicological safety of the final products. The trace and microtrace element contents in the analyzed fermented whey beverages are presented in Table 4.

## 3. Materials and Methods

### 3.1. Materials

For the preparation of fermented organic whey beverages enriched with organic fruit juices, the following materials were used: unpasteurized acid and sweet goat whey, as well as unpasteurized acid and sweet cow whey, all sourced from the Family Organic Farm “Figa” (owned by Waldemar and Tomasz Maziejuk, Mszana, Tylawa, Poland). Organic sea buckthorn and rosehip (*R. canina*) juices were obtained from Polska Róża–Ernest Michalski Ltd. (Falenty Nowe, Raszyn, Poland). The fermentation process was initiated using lyophilized direct-vat-inoculation starter cultures Lactoferm ABY Pro-Tek (Biochem s.r.l., Via La Rinascita, Birori, Nuoro, Italy), containing *Streptococcus thermophilus*, *Bifidobacterium bifidum*, *Lactobacillus acidophilus*, and *Lactobacillus delbrueckii* subsp. *bulgaricus*. Organic brown cane sugar from certified organic farming was supplied by Bio Planet Sp. z o.o. (Leszno, Poland).

### 3.2. Production Process

In the initial stage of production, the ingredients listed in Table 1 were precisely measured to achieve a whey-to-juice ratio of 1:1, using either cow or goat whey (sweet or acid) and sea buckthorn or rosehip (*R. canina*) juice. The whey-to-fruit juice ratio (1:1) was adopted in accordance with our previously established technological protocol for organic fermented whey beverages enriched with sea buckthorn or rosehip juice. This ratio was selected to integrate the nutritional and technological attributes of the whey matrix with a substantial contribution of bioactive compounds derived from the fruit component. Organic brown cane sugar was added only when required to adjust the total sugar content of the formulation to 5%, taking into account the naturally occurring sugars in both whey and fruit juice. This standardization aimed to facilitate lactic acid fermentation and ensure consistent fermentation conditions across all beverage variants. The respective liquids were blended under laboratory conditions at 140 rpm for 30 s with a Heidolph MR 3002S magnetic stirrer (Heidolph Instruments GmbH & Co. KG, Schwabach, Germany) to ensure homogeneity, and the obtained mixture was subsequently passed through a sieve with a pore size of 0.3 mm (approximately 35 gsm) to remove coarse particles. Following filtration, a lyophilized lactic acid starter culture was added at a concentration of 10 g per 100 L of the whey–juice mixture or its aqueous extract. Organic brown cane sugar was then incorporated to reach 5% of the total formulation. The whey-to-fruit juice ratio (1:1), starter culture dosage (10 g/100 L), and fermentation and maturation conditions were defined based on our previously published technological protocol for organic fermented whey beverages enriched with sea buckthorn or rosehip juice. Organic brown cane sugar was incorporated only when necessary to standardize the total sugar content of the formulation at 5%, considering the intrinsic sugars present in both whey and fruit juice. The starter culture suspension was obtained by rehydrating the lyophilized direct-vat inoculum in sterile distilled water prior to its addition to the whey–juice system, ensuring homogeneous distribution of the microorganisms within the matrix. The resulting mixture was then homogenized at 140 rpm for 30 s using a Heidolph MR 3002S magnetic stirrer (Heidolph Instruments GmbH & Co. KG, Schwabach, Germany). This mild homogenization step, performed under laboratory conditions, enabled uniform dispersion of the fruit juice, sugar, and starter culture throughout the whey matrix, while limiting aeration and preventing structural disruption of the beverage. Fermentation was initiated by inoculating the whey–juice mixture with the lyophilized direct-vat starter culture Lactoferm ABY Pro-Tek, comprising *Streptococcus thermophilus*, *Bifidobacterium bifidum*, *Lactobacillus acidophilus*, and *Lactobacillus delbrueckii* subsp. *bulgaricus*. Following inoculation and homogenization, the beverages were incubated at 42 °C for 4 h to facilitate lactic acid fermentation. The samples were then cooled and stored at 5 °C for a 14-day maturation period prior to conducting analyses of chemical composition, mineral and trace elements, fatty acid profile, total polyphenol content, and antioxidant activity. Finally, sterilized bottles were filled with the prepared mixture, sealed, and incubated for 4 h at 42 °C, followed by maturation at 5 °C for 14 days prior to analytical determinations. The sample codes used in this study are presented in Table 5.

### 3.3. Determination of Antioxidant Properties by the DPPH Method

The antioxidant activity of the fermented whey beverages was assessed using the 2,2-diphenyl-1-picrylhydrazyl (DPPH) radical scavenging assay according to Szafrańska et al. [10], with minor modifications. A 0.2 mL aliquot of each sample was combined with an ethanolic solution of 0.1 mM DPPH (Sigma-Aldrich, St. Louis, MO, USA). The mixture was vortexed thoroughly and left to react for 15 min in darkness at room temperature to prevent photodegradation of the reagent. Absorbance values were recorded at 520 nm using a UV–VIS spectrophotometer (Evolution 201 UV–Visible Spectrophotometer, Thermo Fisher Scientific, Waltham, MA, USA). l-Ascorbic acid served as a reference antioxidant. The scavenging activity of the samples was calculated according to the following equation:

DPPH radical-scavenging activity (%) = [1 − (sample absorbance at 520 nm/control absorbance at 520 nm)] × 100

### 3.4. Determination of Antioxidant Properties by the FRAP Method

The ferric reducing antioxidant power (FRAP) of the beverages was evaluated following the method proposed by Benzie and Strain [38], with minor modifications. The FRAP reagent was freshly prepared by mixing 300 mM acetate buffer (pH 3.6) with a 10 mM solution of 2,4,6-tris(2-pyridyl)-1,3,5-triazine (TPTZ; Sigma-Aldrich, St. Louis, MO, USA) in 40 mM HCl, and 20 mM FeCl_3_·6H_2_O (POCH, Gliwice, Poland) in a 10:1:1 volume ratio. A 0.1 mL aliquot of each beverage sample was added to 1.9 mL of the freshly prepared FRAP reagent. The mixture was vortexed and incubated for 15 min at 37 °C to ensure reaction completion. Absorbance readings were recorded at 593 nm using a UV–VIS spectrophotometer. Results are expressed as the concentration of Fe^2+^ ions generated by the reduction of Fe^3+^, indicating the sample’s total reducing power.

### 3.5. Determination of Antioxidant Properties by the ABTS Method

The total antioxidant capacity of the samples was determined using the 2,2′-azino-bis(3-ethylbenzothiazoline-6-sulfonic acid) (ABTS) radical cation decolorization assay according to Szafrańska et al. [10], with minor modifications. The working ABTS solution was prepared by mixing 1 mL of ABTS reagent with 80 mL of methanol and adjusting its absorbance to 0.700 ± 0.002 at 734 nm using a UV–VIS spectrophotometer. To initiate the reaction, 30 μL of each fermented whey beverage was added to 3 mL of the ABTS solution. Blanks were prepared in parallel by replacing the sample volume with methanol. The reaction mixtures were kept in the dark for 5 min at room temperature, after which the decrease in absorbance at 734 nm was recorded. The antioxidant activity is expressed as the percentage of ABTS radical inhibition relative to the control.

### 3.6. Determination of Total Polyphenols (Folin–Ciocalteu Method)

Total polyphenol content was determined using the Folin–Ciocalteu colorimetric method according to Pérez et al. [39], with minor modifications. Methanolic extracts of the samples were combined with Folin–Ciocalteu reagent, followed by sodium carbonate solution. After a 30-min incubation, absorbance was read at 725 nm using a Shimadzu UV-1800 (Shimadzu Corporation, Kyoto, Japan). Quantification was performed using caffeic acid calibration solutions, and results are expressed as mg CAE/100 g.

### 3.7. Determination of Fat Content (Soxhlet Method)

The fat content of the samples was determined using solvent extraction in accordance with the Soxhlet gravimetric procedure based on AOAC methods [40]. Homogenized material was weighed into cellulose extraction thimbles and subjected to hexane extraction using hexane (Avantor Performance Materials Poland S.A., Gliwice, Poland) in a Soxtec Avanti^®^ system (Tecator AB, Höganäs, Sweden). Following the completion of the boiling and rinsing phases, the solvent was recovered and the extraction cups were dried to constant weight in a MEMMERT oven (Memmert GmbH + Co. KG, Schwabach, Germany). Fat content was calculated gravimetrically and is expressed as g/100 g of sample.

### 3.8. Preparation of Fatty Acid Methyl Esters (FAME)

Fatty acid methyl esters were prepared from approximately 100 mg of extracted fat according to ISO 12966-2:2017 [41], with minor modifications. Samples were subjected to saponification with methanolic KOH and subsequently esterified using boron trifluoride in methanol. After heating in a water bath and rapid cooling, FAMEs were extracted into hexane, washed with saturated sodium chloride solution, dried over anhydrous sodium sulfate, and transferred to chromatographic vials. All reagents were obtained from Avantor Performance Materials Poland S.A. (Gliwice, Poland), according to the analytical protocol used in the laboratory.

### 3.9. Gas Chromatographic Analysis of Fatty Acids

FAMEs were analyzed using a Varian 450-GC gas chromatograph, equipped with a CP-8400 autosampler, a 1177 split/splitless injector, a Select™ Biodiesel for FAME capillary column, and a flame ionization detector (Varian Inc., Palo Alto, CA, USA), according to ISO 12966-4:2015 [42], with minor modifications. Helium served as the carrier gas. Chromatograms were processed using the Galaxie™ Chromatography Data System, and fatty acids were identified and quantified using certified FAME reference standards.

### 3.10. Determination of Mineral and Trace Elements (ICP–MS)

Mineral and trace element contents were assessed using inductively coupled plasma mass spectrometry (ICP–MS). Approximately 0.5 g of homogenized sample was weighed with 0.0001 g accuracy and mineralized with Suprapur HNO_3_ (Merck KGaA, Darmstadt, Germany) in a CEM Mars Xpress microwave digestion system (CEM Corporation, Matthews, NC, USA) at 210 °C. The resulting digests were diluted to 50 mL with demineralized water (0.055 µS/cm) and analyzed using a Varian MS-820 ICP–MS (Varian Inc., Palo Alto, CA, USA) under standard argon plasma conditions. Quantification was based on external calibration with Ultra Scientific standards (Ultra Scientific, North Kingstown, RI, USA), and analytical performance was verified using blanks, duplicates, and certified reference material NIST-1577c [43,44].

### 3.11. Determination of Mineral Elements (FAAS)

Selected macro- and microelements were determined by flame atomic absorption spectrometry (FAAS) following the same mineralization protocol as for ICP–MS and according to validated procedures for mineral and trace element determination in dairy matrices [43], with minor modifications. Measurements were performed using a Varian SpectrAA 280 FS spectrometer (Varian Inc., Palo Alto, CA, USA) under element-specific instrumental conditions with an acetylene–air flame. Schinkel buffer was added to minimize ionization interferences. Calibration curves were prepared using certified standard solutions, and results are expressed as mg/kg.

### 3.12. Determination of Mercury Content (AMA 254 Analyzer)

Mercury was determined directly using an AMA 254 mercury analyzer (Altec Ltd., Prague, Czech Republic) according to EPA Method 7473 [45], with minor modifications. Samples were thermally decomposed in a combustion furnace, and the released mercury vapor was trapped on a gold amalgamator. Subsequent thermal desorption allowed quantification at 253.64 nm by atomic absorption. The method required no wet mineralization and was applied to both solid and liquid samples.

### 3.13. Statistical Analysis

The obtained results were subjected to a three-way analysis of variance (ANOVA) (whey origin, whey type, juice type) and differences between mean values were evaluated using Tukey’s post hoc test at a significance level of *p* ≤ 0.05. All statistical analyses were performed using Statistica PL version 13 (StatSoft, Krakow, Poland).

## 4. Conclusions

The present study demonstrates that fermented whey beverages produced from organic goat and cow whey and supplemented with *R. canina* and *H. rhamnoides* juices are valuable functional products characterized by high antioxidant potential, favorable fatty acid composition, and safe levels of trace and heavy metals. Enrichment with *R. canina* juice markedly enhanced the antioxidant capacity of the fermented whey beverages, as indicated by elevated DPPH, ABTS, and FRAP values, along with increased total polyphenol content. This improvement is attributed to the rich composition of phenolic acids, flavonoids, carotenoids, and vitamin C in rosehip, which collectively contribute to strong radical-scavenging and reducing activities. In contrast, supplementation with *H. rhamnoides* juice produced beverages exhibiting more balanced antioxidant profiles and notably high FRAP activity, consistent with the complex mixture of carotenoids and polyphenols naturally present in sea buckthorn fruit. Fatty acid profile analysis indicates that both plant-based additives affect the lipid composition of fermented whey beverages. The introduction of plant-derived lipid fractions from rosehip and sea buckthorn juices may partly compensate for the naturally low fat content of whey, contributing to a more complete nutritional profile compared with plain fermented whey beverages. Rosehip fortification enhanced the content of polyunsaturated fatty acids (PUFAs), particularly linoleic (C18:2 n−6) and α-linolenic (C18:3 n−3) acids, and contributed to relatively low n−6/n−3 ratios in selected samples, ranging from 0.67 to 1.57, as shown in Table 3 (Part b). Sea buckthorn supplementation, in turn, increased monounsaturated fatty acid (MUFA) levels—mainly palmitoleic (C16:1 n−7) and oleic (C18:1 n−9) acids—improving both the nutritional and functional quality of the products. Beverages formulated from goat whey contain higher concentrations of short- and medium-chain fatty acids (C4:0–C10:0), which support better digestibility and potential physiological benefits compared with those produced from cow whey. Evaluation of mineral and heavy metal content confirmed that all samples complied with the European Union food safety standards. Essential mineral elements such as Fe, Zn, and Mn predominate, whereas concentrations of toxic elements—As, Cd, Pb, and Hg—remain well below the regulatory limits defined in Commission Regulation (EC) No. 1881/2006 and its amendment (EU) 2023/915. Beverages containing sea buckthorn juice exhibit the highest Fe concentrations, while Mn levels are strongly influenced by the juice type, with the pure rosehip juice showing the highest Mn content.

Overall, the combination of organic whey with *R. canina* and *H. rhamnoides* juices yielded fermented beverages that meet rigorous food safety and quality requirements while displaying improved nutritional and functional attributes. These products can thus be regarded as promising additions to the functional food market, offering natural sources of antioxidants, essential fatty acids, and minerals, all in accordance with current EU food safety and sustainability regulations. The use of organic raw materials, simple formulation, and low toxic element levels support the potential clean-label and organic-oriented positioning of these beverages. The findings further highlight that the valorization of underutilized by-products such as whey through the incorporation of bioactive plant ingredients contributes both to environmental sustainability and the advancement of health-promoting dairy-based beverages.

## Figures and Tables

**Table 1:** Antioxidant properties and polyphenol content of fermented whey beverages obtained from organic goat and cow whey with the addition of sea buckthorn or rosehip (*R. canina*) juices.

Sample	DPPH [% Inhibition]	ABTS [% Inhibition]	FRAP [mmol TE/L]	Polyphenols (mg/100 g)
SKK/DR	80.99 ^e^ ± 0.94	36.40 ^i^ ± 0.44	1.022 ^h^ ± 0.018	351.7 ^c^ ± 1.91
SKK/NP	61.57 ^d^ ± 0.63	45.14 ^k^ ± 0.15	0.761 ^e^ ± 0.012	21.2 ^a^ ± 1.50
SKK/R	41.52 ^a^ ± 0.68	14.44 ^c^ ± 0.20	0.599 ^c^ ± 0.009	39.1 ^a^ ± 2.89
SKKr/DR	91.00 ^g^ ± 1.78	34.95 ^h^ ± 0.24	0.979 ^g^ ± 0.011	421.6 ^e^ ± 13.30
SKKr/NP	48.77 ^b^ ± 0.72	13.18 ^b^ ± 0.14	0.449 ^a^ ± 0.004	28.5 ^a^ ± 0.44
SKKr/R	94.62 ^h^ ± 0.66	3.10 ^a^ ± 0.04	0.815 ^f^ ± 0.006	41.9 ^a^ ± 2.81
SSK/DR	49.51 ^b^ ± 0.32	42.05 ^j^ ± 0.61	1.162 ^j^ ± 0.011	327.5 ^b^ ± 19.90
SSK/NP	57.23 ^c^ ± 0.27	29.55 ^f^ ± 0.37	0.691 ^d^ ± 0.002	23.2 ^a^ ± 1.82
SSK/R	43.73 ^a^ ± 0.51	3.01 ^a^ ± 0.02	0.536 ^b^ ± 0.006	41.4 ^a^ ± 1.10
SSKr/DR	92.55 ^g^ ± 1.10	32.87 ^g^ ± 0.18	1.122 ^i^ ± 0.017	379.3 ^d^ ± 8.62
SSKr/NP	58.42 ^c^ ± 0.68	25.88 ^e^ ± 0.42	0.772 ^e^ ± 0.009	26.5 ^a^ ± 1.61
SSKr/R	86.28 ^f^ ± 0.29	19.05 ^d^ ± 0.31	0.519 ^b^ ± 0.005	41.4 ^a^ ± 2.46

^a–k^ Statistical differences in the various columns are indicated by different letters (*p* < 0.05). SKKr/R—unpasteurized acid cow whey with sea buckthorn juice; SSKr/R—unpasteurized sweet cow whey with sea buckthorn juice; SSK/R—unpasteurized sweet goat whey with sea buckthorn juice; SKK/R—unpasteurized acid goat whey with sea buckthorn juice; SKKr/DR—unpasteurized acid cow whey with rosehip juice; SSKr/DR—unpasteurized sweet cow whey with rosehip juice; SSK/DR—unpasteurized sweet goat whey with rosehip juice; SKK/DR—unpasteurized acid goat whey with rosehip juice; SSKr/NP—unpasteurized sweet cow whey; SKKr/NP—unpasteurized acid cow whey; SSK/NP—unpasteurized sweet goat whey; and SKK/NP—unpasteurized acid goat whey.

**Table 2:** Ratios between antioxidant indices (mean values) for fermented whey beverages enriched with *R. canina* and *H. rhamnoides*.

Type of Beverage	DPPH/FRAP (Mean ± SD)	FRAP/ABTS (Mean ± SD)	TPC/DPPH (Mean ± SD)
*R. canina*	74.3 ± 21.9	0.029 ± 0.003	4.9 ± 1.1
*H. rhamnoides*	108.3 ± 43.4	0.127 ± 0.113	0.7 ± 0.3

**Table 3:** (a) Fatty acid composition of fermented whey beverages produced from organic goat and cow whey with the addition of sea buckthorn or rosehip (*R. canina*) juices [g/100 g]. (b) Omega-6, omega-3, and n−6/n−3 ratio in fermented whey beverages and fruit juices.

(a)													
	**SKK/R**	**SKK/DR**	**SSKr/NP**	**SKKr/DR**	**SKKr/NP**	**SSkr/R**	**SKK/NP**	**SSK/NP**	**SSK/DR**	**SKKr/R**	**SSK/R**	**Rosehip Juice**	**Sea Buckthorn Juice**
C4:0	0.125	0.136	0.017	0.059	0.192	0.048	0.513	0.068	0.017	0.023	0.148	0.0007	0.051
C6:0	0.173	0.040	0.027	0.016	0.038	0.040	0.091	0.060	0.015	0.026	0.031	0.0007	0.054
C8:0	0.026	0.039	0.028	0.008	0.019	0.022	0.098	0.033	0.008	0.012	0.030	0.00023	ND
C10:0	0.082	0.115	0.087	0.017	0.041	0.050	0.298	0.073	0.018	0.024	0.088	ND	ND
C11:0	ND	ND	ND	ND	ND	ND	ND	ND	ND	ND	ND	0.00023	ND
C12:0	0.031	0.047	0.034	0.020	0.046	0.055	0.123	0.083	0.020	0.026	0.036	ND	ND
C13:0	ND	ND	ND	ND	ND	ND	ND	ND	ND	ND	ND	0.0053	ND
C14:0	0.099	0.130	0.091	0.067	0.156	0.193	0.321	0.284	0.069	0.101	0.091	ND	ND
C14:1n5	ND	ND	ND	0.004	0.012	0.010	ND	0.016	0.005	0.006	0.010	0.0003	ND
C15:0	0.013	0.012	0.009	0.008	0.022	0.022	0.036	0.039	0.009	0.012	ND	ND	ND
C15:1n5	ND	ND	ND	ND	ND	ND	ND	ND	ND	ND	ND	0.0268	ND
C16:0	0.869	0.385	0.268	0.178	0.421	0.790	0.946	0.754	0.188	0.581	0.536	0.0031	0.222
C16:1n7	0.656	0.007	0.006	0.007	0.011	0.346	0.016	0.033	0.008	0.345	0.304	0.0003	0.237
C17:0	0.006	0.010	0.007	0.003	0.015	0.012	0.030	0.019	0.005	ND	0.005	ND	ND
C17:1n7	ND	ND	0.001	ND	ND	ND	ND	0.005	0.002	ND	ND	0.0111	ND
C18:0	0.187	0.246	0.156	0.080	0.199	0.235	0.562	0.357	0.091	0.133	0.158	0.0238	0.013
C18:1n9c	0.298	0.280	0.200	0.106	0.248	0.371	0.711	0.514	0.124	0.209	0.234	0.0037	0.034
C18:2n6c	0.229	0.065	0.028	0.006	0.028	0.124	0.084	0.067	0.015	0.103	0.105	ND	0.058
C18:3n6	ND	0.024	0.012	ND	ND	ND	ND	ND	ND	ND	ND	0.0021	ND
C18:3n3	0.040	ND	ND	0.009	ND	0.028	0.053	0.046	0.011	0.018	0.021	ND	0.005
C20:0	0.002	ND	0.005	ND	0.023	ND	0.001	ND	ND	ND	0.003	ND	ND
C20:1n15	ND	ND	ND	ND	ND	ND	ND	ND	ND	ND	ND	0.0004	ND
C20:1n9	0.009	ND	ND	ND	ND	ND	ND	ND	ND	ND	0.004	ND	0.001
C20:2n6	ND	ND	ND	ND	0.000	ND	ND	ND	ND	ND	ND	ND	0.004
C20:3n6	ND	ND	ND	ND	ND	ND	ND	ND	ND	ND	ND	ND	ND
C21:0	0.004	ND	0.001	ND	ND	ND	ND	ND	ND	ND	0.001	ND	ND
C20:4n6	ND	ND	ND	ND	0.005	ND	ND	ND	ND	ND	ND	ND	ND
C20:3n3	ND	ND	0.001	ND	ND	ND	ND	ND	ND	ND	ND	0.0007	ND
C20:5n3	ND	ND	0.002	ND	ND	ND	ND	ND	ND	ND	ND	ND	ND
C22:0	ND	ND	ND	ND	ND	ND	ND	ND	ND	ND	ND	ND	ND
C22:1n9	ND	ND	ND	ND	ND	ND	ND	ND	ND	ND	ND	ND	ND
C22:2n6	ND	ND	ND	ND	ND	ND	ND	ND	ND	ND	0.001	0.0006	ND
C23:0	ND	ND	ND	ND	ND	ND	ND	ND	ND	ND	ND	0.0006	ND
C24:0	ND	ND	ND	ND	ND	ND	ND	ND	ND	ND	ND	0.0003	ND
C22:6n3	ND	ND	ND	ND	ND	ND	ND	ND	ND	ND	ND	ND	ND
C24:1n9	ND	ND	ND	ND	ND	ND	ND	ND	ND	ND	ND	ND	ND
SFA	1.491	1.025	0.713	0.396	0.963	1.418	2.505	1.702	0.424	0.916	0.979	0.0506	0.289
MUFA	0.963	0.287	0.207	0.117	0.271	0.727	0.726	0.567	0.138	0.560	0.553	0.0273	0.271
PUFA	0.268	0.089	0.043	0.015	0.063	0.152	0.137	0.114	0.025	0.122	0.127	0.0072	0.066
OMEGA 3	0.040	0.000	0.003	0.009	0.034	0.028	0.053	0.046	0.011	0.018	0.021	0.0028	0.005
OMEGA 6	0.229	0.089	0.040	0.006	0.028	0.124	0.084	0.067	0.015	0.103	0.106	0.0044	0.062
OMEGA 9	0.307	0.280	0.200	0.106	0.248	0.371	0.711	0.514	0.124	0.209	0.238	0.0242	0.035
(b)													
**Sample**			**Omega-6**				**Omega-3**			**n−6/n−3 Ratio**			
SKK/R			0.229				0.040			5.73			
SKK/DR			0.089				0.000			NC			
SSKr/NP			0.040				0.003			13.33			
SKKr/DR			0.006				0.009			0.67			
SKKr/NP			0.028				0.034			0.82			
SSKr/R			0.124				0.028			4.43			
SKK/NP			0.084				0.053			1.58			
SSK/NP			0.067				0.046			1.46			
SSK/DR			0.015				0.011			1.36			
SKKr/R			0.103				0.018			5.72			
SSK/R			0.106				0.021			5.05			
Rosehip juice			0.0044				0.0028			1.57			
Sea buckthorn juice			0.062				0.005			12.40			

ND—not detected, because omega-3 fatty acids were not detected or were equal to zero. Sample codes are explained in Section 3.

**Table 4:** Trace and microtrace element content in fermented whey beverages produced from organic goat and cow whey with the addition of sea buckthorn or rosehip (*R. canina*) juices [mg/kg].

Type of Sample	Ca [mg/kg]	Fe [mg/kg]	Zn [mg/kg]	Mn [mg/kg]	As [mg/kg]	Cd [mg/kg]	Pb [µg/kg]	Se [mg/kg]	Ni [mg/kg]	Co [mg/kg]	Hg [mg/kg]
SKK/R	639	8.76	6.15	1.4	0.012	0.007	0.072	0.274	0.066	0.004	<LOQ = 0.005
SKK/DR	859	0.753	3.65	2.34	0.011	0.004	0.044	0.251	0.007	0.003	<LOQ = 0.005
SSKr/NP	297	<LOQ = 0.1	4.41	<LOQ = 0.005	0.012	<LOQ = 0.001	0.047	0.274	<LOQ = 0.005	0.001	<LOQ = 0.005
SKKr/DR	812	1.88	4.51	2.29	0.011	0.004	0.04	0.24	<LOQ = 0.005	0.003	<LOQ = 0.005
SKKr/NP	961	7.3	5.83	<LOQ = 0.005	0.014	<LOQ = 0.001	0.047	0.27	<LOQ = 0.005	0.004	<LOQ = 0.005
SKkr/R	347	3.78	4.3	0.361	0.007	<LOQ = 0.001	0.032	0.196	0.081	0.002	<LOQ = 0.005
SKK/NP	1050	<LOQ = 0.1	5.63	<LOQ = 0.005	0.016	<LOQ = 0.001	0.058	0.311	<LOQ = 0.005	0.003	<LOQ = 0.005
SSK/NP	312	<LOQ = 0.1	1.57	<LOQ = 0.005	0.063	<LOQ = 0.001	0.072	0.291	<LOQ = 0.005	0.002	<LOQ = 0.005
SSK/DR	322	0.894	2.51	2.59	0.008	0.005	0.05	0.241	<LOQ = 0.005	0.003	<LOQ = 0.005
SKKr/R	562	<LOQ = 0.1	4.43	<LOQ = 0.005	0.011	<LOQ = 0.001	0.054	0.254	0.072	0.003	<LOQ = 0.005
SSK/R	165	4.71	4.44	0.574	0.012	0.007	0.056	0.26	0.061	0.002	<LOQ = 0.005
rosehip (*R. canina*) juice	641	1.59	3.03	6.49	0.009	0.01	0.054	0.237	0.042	0.004	<LOQ = 0.005
Sea buckthorn juice	75.1	13.1	2.64	2.13	0.004	0.016	0.11	0.23	0.218	0.003	<LOQ = 0.005

**Table 5:** Sample codes used for fermented whey beverages produced from organic goat and cow whey with the addition of sea buckthorn or rosehip (*R. canina*) juices.

Whey Origin	Whey Type	Fruit Juice Type	Sample Code
Cow	Sweet	Sea buckthorn	SSKr/R
Cow	Sweet	rosehip (*R. canina*)	SSKr/DR
Cow	Sweet	Control (No Plant)	SSKr/NP
Cow	acid	Sea buckthorn	SKKr/R
Cow	acid	rosehip (*R. canina*)	SKKr/DR
Cow	acid	Control (No Plant)	SKKr/NP
Goat	Sweet	Sea buckthorn	SSK/R
Goat	Sweet	rosehip (*R. canina*)	SSK/DR
Goat	Sweet	Control (No Plant)	SSK/NP
Goat	acid	Sea buckthorn	SKK/R
Goat	acid	rosehip (*R. canina*)	SKK/DR
Goat	acid	Control (No Plant)	SKK/NP

## Data Availability

The data presented in this study are available from the corresponding author upon reasonable request.

## Abbreviations

The following abbreviations are used in this manuscript:

ABTS	2,2′-azino-bis(3-ethylbenzothiazoline-6-sulfonic acid)
DPPH	2,2-diphenyl-1-picrylhydrazyl
DW	dry weight
FAAS	flame atomic absorption spectrometry
FAME	fatty acid methyl esters
FRAP	Ferric Reducing Antioxidant Power
ICP–MS	inductively coupled plasma mass spectrometry
LOQ	limit of quantification
MUFA	monounsaturated fatty acids
ND	not detected
NP	No Plant (control sample)
PUFA	polyunsaturated fatty acids
SFA	saturated fatty acids
TE	Trolox equivalent
